# Overcoming the Limitations of Sputtered Nickel Oxide for High‐Efficiency and Large‐Area Perovskite Solar Cells

**DOI:** 10.1002/advs.201700463

**Published:** 2017-10-26

**Authors:** Guijun Li, Yibin Jiang, Sunbin Deng, Alwin Tam, Ping Xu, Man Wong, Hoi‐Sing Kwok

**Affiliations:** ^1^ College of Electronic Science and Technology Shenzhen University 518061 Shenzhen P. R. China; ^2^ State Key Lab on Advanced Displays and Optoelectronics the Hong Kong University of Science & Technology Clear Water Bay Kowloon 999077 Hong Kong

**Keywords:** large‐scale manufacturing, magnesium doped nickel oxide, magnetron sputtering, perovskite solar cells

## Abstract

Perovskite solar cells (PSCs) are one of the promising photovoltaic technologies for solar electricity generation. NiO*_x_* is an inorganic p‐type semiconductor widely used to address the stability issue of PSCs. Although high efficiency is obtained for the devices employing NiO*_x_* as the hole transport layer, the fabrication methods have yet to be demonstrated for industrially relevant manufacturing of large‐area and high‐performance devices. Here, it is shown that these requirements can be satisfied by using the magnetron sputtering, which is well established in the industry. The limitations of low fill factor and short‐circuit current commonly observed in sputtered NiO*_x_*‐derived PSCs can be overcome through magnesium doping and low oxygen partial pressure deposition. The fabricated PSCs show a high power conversion efficiency of up to 18.5%, along with negligible hysteresis, improved ambient stability, and high reproducibility. In addition, good uniformity is also demonstrated over an area of 100 cm^2^. The simple and well‐established approach constitutes a reliable and scale method paving the way for the commercialization of PSCs.

Lead halide perovskite solar cells (PSCs) were first used as sensitizer in dye‐sensitized solar cells and have attracted enormous attentions as a promising photovoltaic (PV) technology over the last few years.^1^ Nowadays, the most advanced PSCs have a certified high efficiency of 22.1%, making them competitive with crystalline silicon and other leading thin film solar cell technologies.^2^ Such a swift rise of the PSC efficiency has been unprecedented in the history of PV research. The future holds even greater promise for perovskite‐based tandem solar cells, the efficiency of which has been improved by over 26% most recently, and with a possibility of up to 30% in the offing.^3^, ^4^

In general, PSCs can be made with a mesoporous architecture or a planar structure.^5^, ^6^ Despite the mesoporous structures being widely used to deliver top‐performing PSCs with efficiencies larger than 20%,^7^, ^8^, ^9^, ^10^ there is growing interest in the planar architectures, mainly because of their easy fabrication, low‐temperature processing, relatively small hysteresis, and suitability for tandem device application.^11^, ^12^, ^13^, ^14^ Furthermore, the planar heterojunction may possess appealing advantages over the mesoporous structure for up‐scaling manufacturing and improved long‐term device operational stability in terms of the choices of various charge transport materials.^15^ For example, the inorganic hole/electron transporting layers, which have good environmental stability and low cost in comparison with their organic counterparts, can be easily fabricated and employed for planar heterojunction PSCs. Developing inorganic charge transport layers is also considerably important for the continued evolution of high‐performance PSCs that can be used in real‐life applications. In the case of the hole transport layers (HTLs), promising candidates such as Cu_2_O, CuO, CuI, CuSCN, and NiO*_x_* have been widely reported to replace the acidic and hygroscopic PEDOT:PSS in the PSCs.^16^, ^17^ Among these materials, NiO*_x_* has a wide bandgap for the light penetration, an energetically favourable energy level alignment with that of perovskite at the valence band to allow for efficient hole extraction/transport, and a high conduction band energy to effectively block the electron for suppressing the recombination term of the device.

It is well known that NiO*_x_* can be prepared from a number of deposition methods, thereby enabling the versatility of the fabrication process. To date, studies on PSCs using NiO*_x_* as the HTLs have made great progress in their power conversion efficiency (PCE). For example, the solution process, which is desirable for low‐cost and easy production, has been utilized for the preparation of the Li doped Ni*_x_*Mg_1−_ *_x_*O film to achieve >18% efficiency PSCs.^18^, ^19^ And most recently, the efficiency was further improved to 19.19% for a 1.025 cm^2^ perovskite solar cell by the same group.^20^ However, most of the reported solution processes require high‐temperature annealing to convert and crystallize the precursors into polycrystalline nickel oxide. This high‐temperature process makes manufacture more complex and hampers the development of perovskite‐based tandem photovoltaics.^21^, ^22^, ^23^, ^24^ Although low‐temperature solution processes, such as the combustion method^25^ and presynthesized methods have been demonstrated to be able to make PSCs with decent PCEs (>15%),^26^, ^27^, ^28^ industrially relevant fabrication on a large scale and with a high yield is still challenging. Meanwhile, pulse laser deposition and atomic layer deposition have also been reported to deposit the NiO*_x_* for PSCs, demonstrating high efficiencies of 17.3% and 16.4%, respectively.^29^, ^30^ Nevertheless, these methods are expensive, rendering them only suitable for proof‐of‐concept devices. Therefore, there is a pressing need to develop an industrial method for the preparation of charge transport layers that enable low‐cost, large‐scale, and reliable manufacturing of PSCs with high PCEs.

Compared with the above deposition methods, magnetron sputtering is a well‐established and large‐scale deposition technique for making the thin film semiconductors in modern industry. Indeed, sputtered NiO*_x_* HTLs have been reported previously for PSCs. Cui et al. reported PSCs with reactive magnetron sputtering of NiO*_x_* HTL, the PCE of which was only 9.83%;^31^ and Wang et al. demonstrated the sputtered NiO*_x_* as an electron blocking layer in a mesoscopic NiO*_x_*/CH_3_NH_3_PbI_3_ PSC, achieving an efficiency of 11.6%.^32^ The low efficiency of these devices, which arises from the poor fill factor (FF) and the small short‐circuit current (*J*_sc_), largely limits the implementation of this industrial method for large‐scale manufacturing of high‐performance perovskite optoelectronic devices.

In this work, we demonstrate that the limitations of the poor FF and *J*_sc_ in sputtered NiO*_x_*‐derived PSCs can be overcome through magnesium doping and low oxygen partial pressure deposition. The p–i–n PSCs employing the magnesium doped NiO*_x_* (NiMgO*_x_*) HTLs achieve a high efficiency of up to 18.5%, outperforming the previous results. Furthermore, good uniformity is demonstrated for PSCs over a large area of 100 cm^2^, which signifies its general applicability for large‐scale manufacturing. Our work on reactive magnetron sputtered NiO*_x_* represents an important step for upscaling high‐efficiency PSCs to a size suitable for achieving industrially relevant perovskite photovoltaic technology.

NiO*_x_* and NiMgO*_x_* are deposited by the room‐temperature reactive magnetron sputtering. More details of the sputtering process can be found in the Experimental Section. The chemical composition and electronic states of pristine NiO*_x_* and NiMgO*_x_* are investigated with X‐ray photoelectron spectroscopy (XPS). The Mg 2p spectrum is clearly detected at binding energies of 48.7 eV for the NiMgO*_x_* films (Figure S1A, Supporting Information), providing convincing evidence of the existence of Mg in the NiO*_x_* film. The Mg fraction could be relatively controlled with atomic ratios of 2.3%, 4%, 8%, and 12%, by varying the radio‐frequency (RF) powers of the MgO target during the cosputtering process. The Mg content is further determined by energy‐dispersive X‐ray spectroscopy (EDS), and the results from the XPS and EDS measurements are in good agreement (Figure S1B, Supporting Information). The Ni 2p_3/2_ and O 1s characteristic peaks of the films in the XPS spectra are shown in Figure S2 in the Supporting Information. The figure shows that the Ni 2p_3/2_ XPS spectrum can be deconvoluted into three sub‐peaks using the Gaussian function. They correspond to three different binding states, including the Ni metal at the peak position of 852.6 eV and two oxidation states assigned to Ni^2+^ in the standard Ni—O octahedral bonding configuration in NiO, as well as the Ni^2+^ vacancy‐induced Ni^3+^ in Ni_2_O_3._ 33 The O 1s XPS peaks of NiO*_x_* at 529.27 eV as well as at 530.99 eV can also be fitted with the binding states of Ni^2+^ and Ni^3+^. For the NiMgO*_x_* films, there is a slight blue‐shift of the Ni^2+^ and Ni^3+^ peaks with respect to NiO*_x_* in the O 1s XPS spectra (Figure S3, Supporting Information), which is consistent with the literature.^34^ The detailed chemical composition ratio is summarized in **Table** **1**. Since the oxygen partial press during sputtering is as low as 3%, the atomic ratio of oxygen over Ni in the pristine NiO*_x_* is found to be only 0.85. However, the atomic ratio of oxygen increases along with the increasing of the Mg content in the film owing to the rise of the RF power for the MgO target. This finding is thought to be related to reducing of the Ni metal state and increasing of the Ni^3+^, as indicated by the Ni 2p_3/2_ and O 1s XPS spectra. To be more specific, the Ni^3+^/Ni ratio calculated from the O 1s XPS spectra shows that the Ni^3+^/Ni rises from 39% to 49% when the Mg concentration increases from 0% to 12%, revealing that the Mg doping could contribute to the increase of relative content of Ni^3+^ acceptors in the NiMgO*_x_* films, which is consistent with phenomena in Li‐doped NiO*_x_*, where Ni^3+^ acceptors are also increased as a result of Li doping.^35^

**Table 1:** Summary of chemical composition of sputtered NiO*_x_* and NiMgO*_x_* films from XPS measurement

No	Name	Mg/(Ni+Mg) [%]	Ni3+/Ni [from O 1s peak,%]	O/Ni [%]
1	NiO*x*	0	39	0.85
2	Ni_0.977_Mg_0.023_O*_x_*	2.3	43	1.03
3	Ni_0.96_Mg_0.04_O*_x_*	4	44	1.05
4	Ni_0.92_Mg_0.08_O*_x_*	8	48	1.11
5	Ni_0.88_Mg_0.12_O*_x_*	12	49	1.23

It is generally accepted the nickel vacancy, namely the Ni^3+^ acceptor, is a self‐dopant in the NiO, rendering the NiO more conductive.^36^ Conductive atomic force microscopy (c‐AFM) is used to compare the conductivity of the NiO*_x_* and NiMgO*_x_* films. As shown in **Figure** **1**A,B, Ni_0.92_Mg_0.08_O*_x_* has a higher conductivity compared to that of pristine NiO*_x_*. At a bias potential of 0.5 V, the electric current is increased by a factor of ≈4 (from ≈1 pA to ≈4 pA) upon replacing the NiO*_x_* with a Ni_0.92_Mg_0.08_O*_x_* film. The conductivity is therefore increased from 0.67 × 10^−6^ to 0.27 × 10^−5^ S cm^−1^, with almost one order of enhancement. The high conductivity of Ni_0.92_Mg_0.08_O*_x_* originates from the increasing of the Ni^3+^ acceptors. However, this enhancement is not as significant as in the cases of Li or Cu‐doped NiO*_x_*.^37^, ^38^ Figure 1C is the X‐ray diffraction (XRD) of the films. Both the NiO*_x_* and NiMgO*_x_* films exhibit distinct XRD peaks of (110), (200), and (220), representing a rock‐salt cubic structure, and there are no impurity peaks corresponding to the planes of MgO. These typical XRD patterns show grains ≈10–14 nm in diameter, as estimated from full width at half maximum (FWHM) of the (200) diffraction peaks using the Scherrer equation. Particularly, all the films are grown in 200‐orientation, and a slight shift of all the peaks for NiMgO*_x_*, in comparison to that of the NiO*_x_*, is observed due to the existence of Mg. However, excessive Mg (Mg content >12%) will be detrimental to the crystallinity of the film. In addition, we found all the films show good transparency, which may be ascribed to the relatively low concentration of Ni^3+^ in the film.^39^ In particular, as shown in Figure 1D, NiMgO*_x_* shows a slightly higher transmittance at short wavelength (<500 nm). This is different from the Li or Cu‐doped NiO*_x_*, where the transmittance decreases upon doping.^37^, ^40^

**Figure 1:**
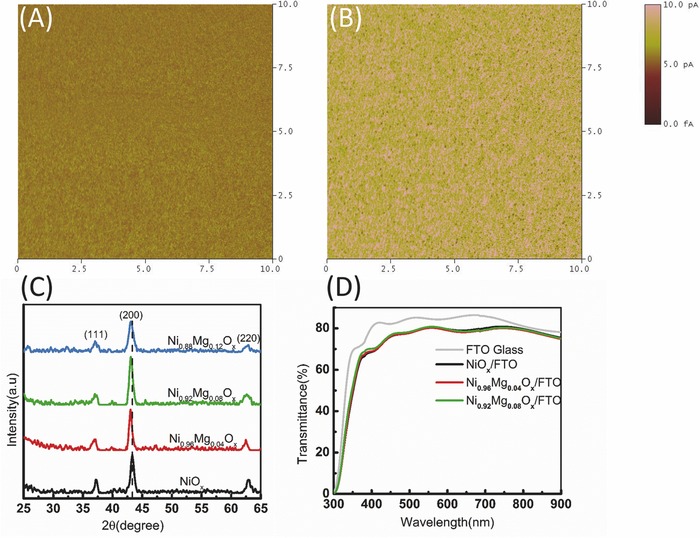
Comparison of the conductivity mapping results for A) NiO*_x_*, and B) Ni_0.92_Mg_0.08_O*_x_*. C) Comparison of the XRD pattern of NiO*_x_* and NiMgO*_x_*. D) Comparison of the transmittance of NiO*_x_* and NiMgO*_x_*, the FTO transmittance is given as reference.

In a solar cell structure, the energy band alignment as well as the interface between the charge transport layers and the perovskite absorbing layer play critical roles in the charge extraction/transfer process. We have characterized the energy band diagrams of NiO*_x_* and NiMgO*_x_* with ultraviolet photoelectron spectroscopy (UPS). The work function and the difference between the valence band and Fermi level (*E*_v_ − *E*_F_) are shown in **Figure** **2**A. As the Mg content is increased, the valence band maximum (VBM) of the film continues to shift downward. A maximum value of 0.2 eV for the VBM downshift is achieved when compared with the NiO*_x_*. A deeper VBM of the HTL, as shown in the energy band diagram of the PSCs in Figure 2B, can well match the VBM of the perovskite to minimize the energy redundancy for the holes injection. The reduced energy band offset is expected to benefit the holes transfer, increase the device photovoltage as well as other photovoltaic parameters.^41^

**Figure 2:**
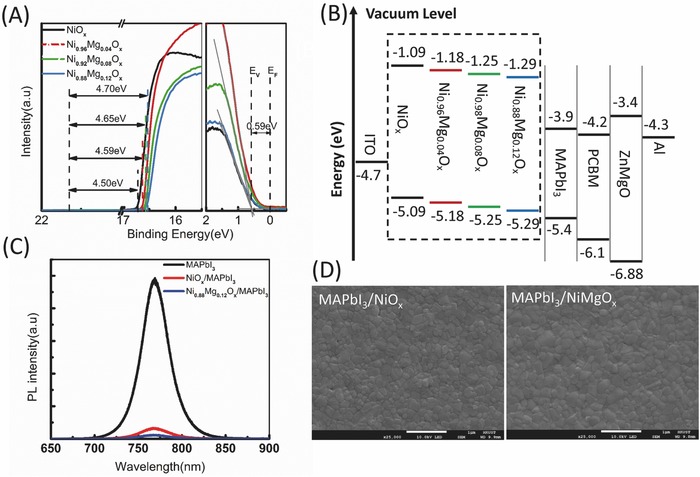
A) UPS (He I) spectra for the NiO*_x_* and NiMgO*_x_* thin films. The spectra give the information of (left panel) the photoemission cutoff energy and (right panel) the energy difference between the valence band maximum and the Fermi level. The work function is given by *W* = 21.22 eV (cutoff energy). B) Energy‐level diagrams of the investigated NiO*_x_* and NiMgO*_x_* in a perovskite solar cell structure; the energy bandgap of NiO*_x_* and NiMgO*_x_* thin films are obtained with a value of about 4 eV using the Tauc plot method. C) Photoluminescence spectrum of CH_3_NH_3_PbI_3_ (MAPbI_3_) deposited on glass (black), NiO*_x_* (red) and Ni_0.92_Mg_0.08_O*_x_* (blue), respectively. D) Scanning electron microscopy (SEM) images of CH_3_NH_3_PbI_3_ films deposited on NiO*_x_* (left) and Ni_0.92_Mg_0.08_O*_x_*(right), respectively.

The charge transfer process between the perovskite and HTL is also dominated by the perovskite/HTL interface, which can be analyzed by the steady‐state photoluminescence (PL) measurement, as shown in Figure 2C. Apparently, the Ni_0.92_Mg_0.08_O*_x_* shows a significant PL quenching compared with that of the NiO*_x_*, indicating holes transfer from perovskite to Ni_0.92_Mg_0.08_O*_x_* HTL occurs effectively. This fast charge transfer is needed to enhance the charge decay channel to remove the excess holes in the perovskite absorbing layer to prevent possible recombination, which is believed to deliver high‐performance PSCs without hysteresis.

PSCs with different HTLs are fabricated. First, the fluorine‐doped tin oxide (FTO) glass is patterned by laser scribing, followed by reactive sputtering of NiO*_x_*, and NiMgO*_x_* is obtained via the cosputtering of the ceramic MgO target and the metallic nickel target. The CH_3_NH_3_PbI_3_ light absorbing layer, with a thickness of around 300 nm, is deposited by a vacuum‐assistant solvent‐engineering process, and then a thin phenyl‐C61‐butyric acid methyl ester (PCBM) layer and an n‐type ZnMgO layer are sequentially deposited by spin coating from a chlorobenzene solution and an ethanol solution, respectively. Finally, the device is completed by thermal evaporation of a 100 nm Al cathode. Figure 2D depicts the surface morphology of the CH_3_NH_3_PbI_3_ deposited onto each of the NiO*_x_* and NiMgO*_x_*, suggesting there is no apparent difference between them.

The resultant *J*–*V* curves of the PSCs with different HTLs under AM 1.5 G conditions are shown in **Figure** **3**A, and the relevant solar cells parameters are summarized in **Table** **2**. The NiO*_x_*‐based PSCs exhibit a relatively low PCE of 14.7%, along with a *V*_oc_ of 1041 mV, a FF of 0.7, and a *J*_sc_ of 20.3 mA cm^−2^. Despite this, it is worthwhile noting that our sputtered NiO*_x_*‐based solar cells still perform better than the previously reported results, the highest efficiency of which is only 11.6%.^31^, ^32^ We note that the previous results are based on the NiO*_x_* HTL sputtered using a high oxygen partial pressure. It has been confirmed that, as shown in Figure S4A in the Supporting Information, the solar cells with NiO*_x_* sputtered using a low oxygen partial pressure have a higher performance than those of the devices with NiO*_x_* sputtered using a high oxygen partial pressure. A possible reason is that the presence of excess Ni^3+^ in the film sputtered from a high oxygen partial pressure may be responsible for the high rate of recombination, which has also been widely reported in the dye‐sensitized solar cells previously.^42^, ^43^, ^44^ However, further explanation of this improvement is needed, which is out of the scope of this paper.

**Figure 3:**
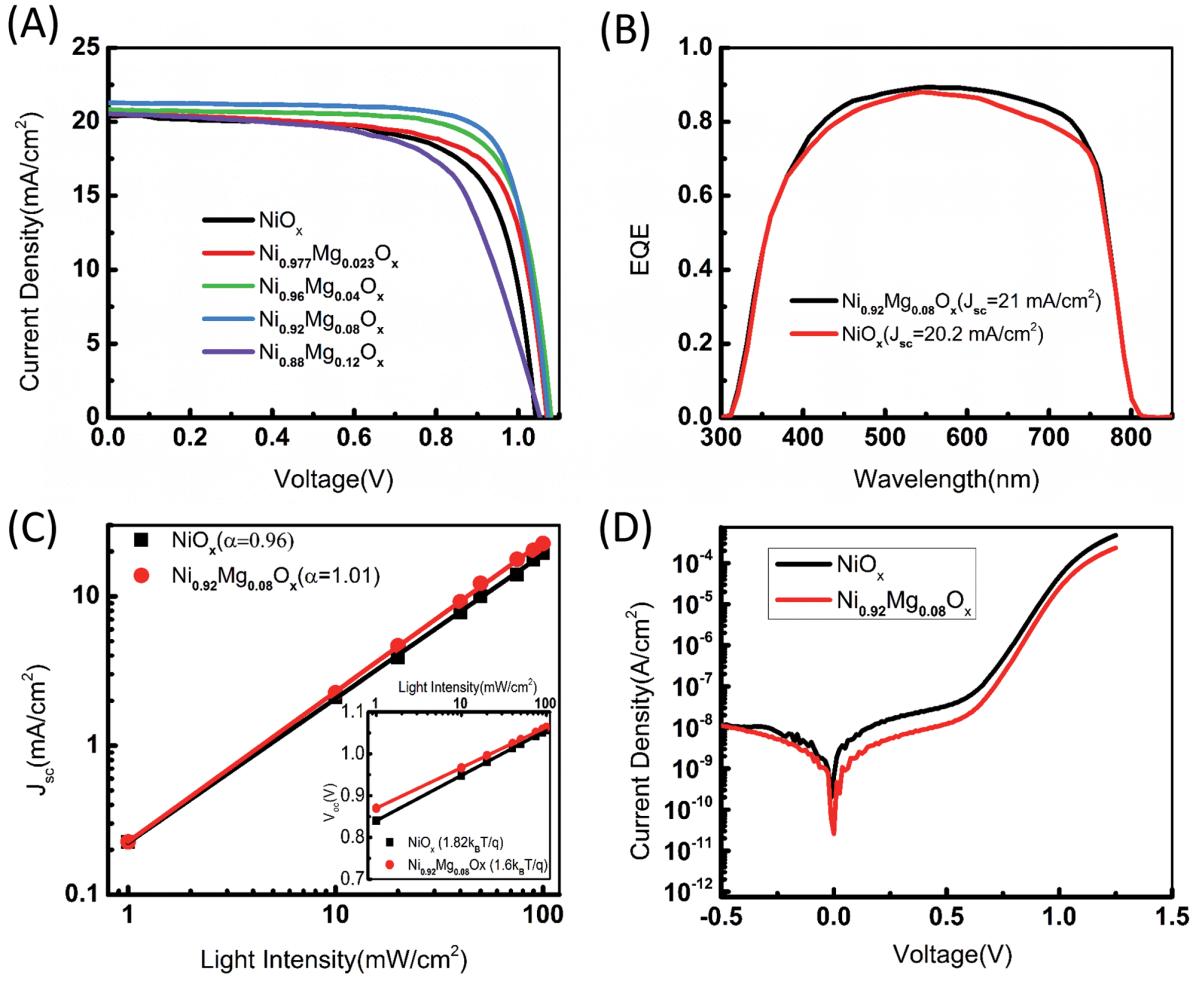
A) *J*–*V* curves of the PSCs with different HTLs. B) External quantum efficiency (EQE) of the PSCs with different HTLs. C) *J*_sc_ dependence and *V*_oc_ dependence (inset) upon different light intensities. D) Dark current–voltage curves of PSCs with NiO*_x_* and NiMgO*_x_* HTLs.

**Table 2:** The relevant device parameters of the PSCs with different HTLs. Each value is obtained from an average of eight cells

HTLs	*V*_oc_ [mV]	*J*_sc_ [mA cm^−2^]	FF [%]	PCE [%]	*R*_s_ [Ω cm^2^]	*R*_sh_ [Ω cm^2^]
NiOx	1041	20.3	70	14.7	4.7	890
Ni_0.977_Mg_0.023_O*_x_*	1069	20.5	72	15.8	5.2	1023
Ni_0.96_Mg_0.04_O*_x_*	1083	20.8	76	17.1	4.9	3069
Ni_0.92_Mg_0.08_O*_x_*	1078	21.3	79	18.2	5.0	5108
Ni_0.88_Mg_0.12_O*_x_*	1053	20.5	64	13.8	13	978

As expected, PSCs exhibit an impressive improvement of the PCE when the Mg is introduced in NiO*_x_*. The highest average PCE of 18.2% is obtained with the Mg content of 8%, and further improvement in the Mg content to 12% is detrimental to the device. The champion cell has a peak PCE of 18.5%, a *V*_oc_ of 1083 mV, a *J*_sc_ of 21.3 mA cm^−2^, and a FF of 0.80 (Figure S4B, Supporting Information). The major improvement lies in the increased FF (from 0.70 to 0.79), which can be attributed to the improvement of the shunt resistance as indicated in Table 2. The large shunt resistance in the NiMgO*_x_*‐derived solar cells is owing to the better energy alignment at the perovskite/HTL interface to allow efficient holes extraction, and significant PL quenching to suppress the recombination. In addition to the FF, *V*_oc_, and *J*_sc_ are also slightly improved in the NiMgO*_x_*‐based PSCs, which can be interpreted as the result of minimized potential loss and efficient carrier collection. Meanwhile, note that the high transmittance of NiMgO*_x_* at the short wavelength is also partly attributed to the improved *J*_sc_. The external quantum efficiency (EQE) shown in Figure 3B indicates better photo‐to‐electron conversion efficiency in NiMgO*_x_*‐based solar cells. The integrated photocurrent (21 mA cm^−2^ for Ni_0.92_Mg_0.08_O*_x_* and 20.2 mA cm^−2^ for NiO*_x_*) from the EQE spectra matches well with the *J*–*V* results.

Figure 3C is the light intensity dependence of the *J*–*V* characteristics of the PSCs used to investigate the recombination mechanisms that dominate the device performance. According to previous studies, *J*_sc_ follows a power law dependence of the light intensity. The power value for the NiMgO*_x_*‐based device (1.01) is higher than that of the NiO*_x_*‐based device (0.96), implying that NiMgO*_x_* mitigates the interfacial recombination loss at the perovskite/HTL interface as a result of small energy band offset.^45^ Under open‐circuit voltage condition, the photogenerated electrons and holes will eventually recombine through shallow traps at the interface or through the impurities and defects in the photoactive layer. The *V*_oc_ dependence of the light intensity, therefore, can then be used to identify the precise recombination pathway in the device. A weaker *V*_oc_ dependence on the light intensity of the NiMgO*_x_*‐based device (with a slope of 1.6*k*_B_ *T*/*q*) compared to that of the NiO*_x_*‐based device (with a slope of 1.82*k*_B_ *T*/*q*) suggests the reduced trap‐assisted Shockley−Read−Hall (SRH) recombination. Since all the devices have the same structure except for the perovskite/HTL interface, the reduced interfacial trap‐assisted recombination can also be ascribed to the improved perovskite/NiMgO*_x_* interface facilitating charge collection and transport.

Dark *J*–*V* characteristics measurement is an effective way to gain insight into the diode behavior of the device with the saturation current (*j*_o_) and the ideal factor (*n*).^46^, ^47^ The dark current–voltage curves of PSCs with NiO*_x_* and NiMgO*_x_* HTLs are given in Figure 3D. The NiMgO*_x_*‐based device possesses a lower saturation current (*j*_o_ = 1.0 × 10^−9^ mA cm^−2^) and ideal factor (*n* = 1.62) compared to that of the NiO*_x_*‐based device (*j*_o_ = 1.6 × 10^−10^ mA cm^−2^, *n* = 1.76), affirming the reduced recombination and much better hole selectivity in NiMgO*_x_*‐derived PSCs.

The current–voltage hysteresis has been widely observed in PSCs. **Figure** **4**A shows the forward and reverse scanning *J*–*V* curves of one of the high‐performance NiMgO*_x_*‐based devices. There is a negligible discrepancy between different scan directions. The scan rates dependence of the *J*–*V* curves is also given in Figure S5 in the Supporting Information, suggesting a small hysteresis for our devices. This is consistent with inverted‐structured perovskite solar cells that usually exhibiting smaller hysteresis than other device structures, owing to the balance of the large amount of photogenerated electrons and holes.^18^, ^19^ The stabilized photocurrent and PCE (Figure 4B) also support the hysteresis‐free behavior of our devices based on NiMgO*_x_* HTL. Additionally, the resulting Ni_0.92_Mg_0.08_O*_x_*‐based devices demonstrate good ambient stability over a period of 600 h at humidity of 50–70%, and maintain 90% of its initial efficiency (Figure S6, Supporting Information).

**Figure 4:**
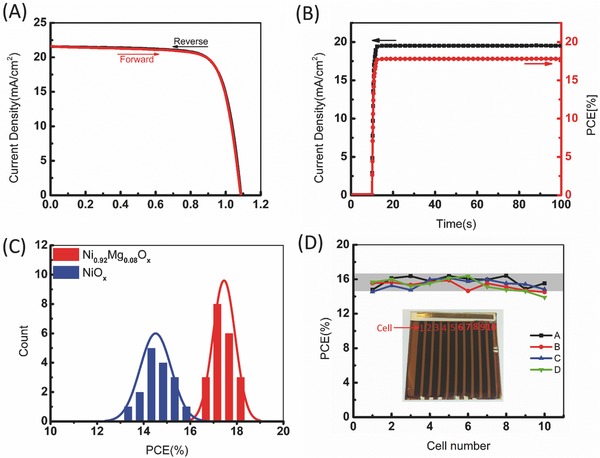
A) *J*–*V* curves for one of the Ni_0.92_Mg_0.08_O*_x_* PSCs measured by forward and reverse scans, showing negligible hysteresis. The delay time is set at 150 ms for both scan directions. B) Steady‐state photocurrent measured at a bias voltage (0.912 V) near the maximum power point and stabilized power output. C) Histogram of PSCs with NiO*_x_* and Ni_0.92_Mg_0.08_O*_x_* HTLs, efficiencies are measured using forward scans at a delay time of 150 ms. The lines are a fitting of the distribution PCEs. D) PCE distribution of the PSCs. Panels (A), (B), (C), and (D) are the four subsubstrates divided from a 10 cm × 10 cm substrate with 30 nm Ni_0.92_Mg_0.08_O*_x_* coated. Each subsubstrate, as shown in the inset, has 10 cells, each cell has an area of 1 cm^2^.

Successful large‐scale manufacturing of the PSCs relies on fabrication reproducibility and large‐area uniformity. One of the advantages of magnetron sputtering compared to other processes is its high yield, which benefits from the extensive experience obtained from the displays, solar cells, and other thin film coating industries. Figure 4C shows the histogram of PCEs for several batches of 24 Ni_0.92_Mg_0.08_O*_x_*‐based devices and 16 NiO*_x_*‐based devices. The PCEs of Ni_0.92_Mg_0.08_O*_x_*‐based PSCs fluctuate within a narrow range between 16.5% and 18.5%, with an average value of 17.5%, whereas the PCEs of NiO*_x_*‐based devices show a range between 13% and 16%, with an average value of 15%. Such a high reproducibility comes from the good uniformity of each layer. The double electron transport layer of PCBM/ZnMgO also helps to prevent possible shunting created by the back contact metal.

In order to verify the large‐area uniformity of the sputtered NiMgO*_x_*, a 10 cm × 10 cm FTO substrate was used. The appearance of the substrate coated with NiMgO*_x_* looks homogeneous in a size of 100 cm^2^ (Figure S7, Supporting Information). Because it was limited by the spin‐coating process for deposition of the perovskite and subsequential layers in our lab, the 10 cm × 10 cm substrate was divided into four subsubstrates (identified as A, B, C, and D) to make the PSCs. For each subsubstrate, there were 10 cells, each with an area of 1 cm^2^ defined by the Al electrode. From Figure 4D we can see that the PCEs show good uniformity not only for each subsubstrate but also from one subsubstrate to another (*V*_oc_, *J*_sc_, and FF are given in Figure S8 in the Supporting Information). An average efficiency of 15.6% is obtained for these 1 cm^2^ cells distributed over the 10 cm × 10 cm substrate, along with a small deviation. A typical *J*–*V* curve for the solar cell from the 10 cm × 10 cm substrate (with a cell area of 1 cm^2^) is plotted with respect to the small cell (0.1 cm^2^), as shown in Figure S9 in the Supporting Information. The low PCE of the large‐area cell is primarily due to the poor FF caused by large series resistance as indicated from the slope near open‐circuit voltage, which is thought to be related to the device area. Further efficiency improvement is anticipated to when a mixed perovskite absorber is employed in the near future.

In summary, we have proposed a strategy to overcome the limitations of low FF and *J*_sc_ commonly observed in PSCs with a magnetron sputtered NiO*_x_* HTL, by introducing magnesium doping and depositing at a low oxygen partial pressure deposition condition. The effect of the magnesium in the NiO*_x_* HTL is carefully studied, revealing that the NiMgO*_x_* film has better transmittance and conductivity. In addition, the NiMgO*_x_* used as a HTL in the PSC also demonstrates a better energy band alignment and pronounced PL quenching, which is critical to reducing potential loss during hole injecting and for efficient charge collection/transport. As expected, the NiMgO*_x_*‐based PSCs show improved PCE compared to that of the NiO*_x_*‐based devices, mainly lying in the large improvement of the FF. Furthermore, the *V*_oc_ and *J*_sc_ are also improved. The optimum magnesium content is found to be 8%. The PSCs with such a NiMgO*_x_* HTL demonstrate the highest PCE of up to 18.5%, along with negligible hysteresis, improved ambient stability, and high reproducibility. The improvement of the PCE is further investigated by using the light intensity dependent *J*–*V* characteristics measurement and the dark *J*–*V* measurement, both of which imply the reduced recombination and much better holes selectivity in NiMgO*_x_*‐based devices. Our devices also show good uniformity over a large area of 10 cm × 10 cm, paving the way for commercialization of PSCs by using this industrial deposition method.

## Experimental Section

*NiO_x_ and NiMgO_x_ Films Preparation*: NiO*_x_* was deposited by room temperature magnetron sputtering (AJA) through a metallic nickel target (purity: 99.9%), in an atmosphere of Ar/O_2_. During the process, the pressure of the chamber was kept at 5 mbar and the DC power was kept at 80 W, yielding a deposition rate of around 6 nm min^−1^. The NiMgO*_x_* was cosputtered from the metallic nickel target and the ceramic MgO target. The other process parameters were the same as those for NiO*_x_*. The Mg concentration was controlled by varying the RF power of the MgO target during the sputtering process. After sputtering, all the films were postannealed in air at a temperature of 300 °C for 1 h.

*Perovskite Solar Cells Fabrication*: FTO substrates were first patterned by the laser scribing, and then cleaned with a detergent solution and rinsed. Second, the UV–ozone treatment was done prior to the sputtering, and a 30 nm NiO*_x_* or NiMgO*_x_* was then sputtered onto the cleaned FTO substrate, followed by a postannealing at 300 °C for 1 h in air. After cooling down to room temperature, the substrate was transferred into the glove box to deposit the perovskite absorbing layer. The CH_3_NH_3_PbI_3_ perovskite solution was prepared from a precursor solution made of PbI_2_ (1.2 m, Sigma‐Aldrich, 99%) and MAI (1.2 m, Dyesol) in a mixture of GBL:DMSO (v:v, 7:3). The perovskite solution was then spin coated onto the HTL at 1000 rpm for 5 s and 6000 rpm for 10 s. After the spin coating, the substrate was immediately put in a closed chamber, pumped down to a pressure of 20 mtorr and then vented to the atmosphere. After annealing of the perovskite layer on a hot plate at 100 °C for 10 min, the PCBM and ZnMgO were then spin coated onto the perovskite layer acting as the electron transport layer, from a chlorobenzene solution (20 mg mL^−1^) and an ethanol solution (10 mg mL^−1^), respectively. Finally, the device was completed by thermal evaporation of a 100 nm Al cathode. For the large‐area fabrication, the same procedure was used, except for the pattern design and the shadow mask used for the Al electrode evaporation.

*Characterization*: X‐ray diffraction patterns were measured using an X‐ray diffractometer (Panalytical X'Pert Pro), using Cu‐Kα radiation (λ = 1.54050 Å). Scanning electron microscopy (SEM) images were obtained using an analytical field emission SEM (JEOL‐7100F). AFM images were performed using a Digital Instruments Dimension 5000 scanning probe microscope in “conductive” mode. The optical measurement was carried out on a Perkin–Elmer spectrophotometer (model Lambda 20). The PL measurement was conducted with a Renishaw MicroRaman/photoluminescence system, for which a He‐Ne laser with 632.8 nm was used as the excitation light source. Current–voltage characteristics were measured under simulated AM1.5G sunlight at 100 mW cm^−2^ irradiance, generated by a light source of a 450 W xenon lamp (Oriel, Sol2A). The light intensity was calibrated using an NREL calibrated Si reference cell. A metal mask was used to reduce the influence of the scattered light. A light intensity dependence *J*–*V* curve was obtained by using neutral filters. And dark *J*–*V* curve was measured by using an IV probe station in a dark light condition. The EQE spectra performed here were obtained from an incident photon‐to‐current efficiency (IPCE) setup consisting of a xenon lamp (Oriel, 450 W) as the light source, a monochromator, a chopper with a frequency of 100 Hz, a lock‐in amplifier (SR830, Stanford Research Corp), and a Si‐based diode for calibration.

## Conflict of Interest

The authors declare no conflict of interest.

## Supporting information

Supplementary
