# Supplementary material for: Overcoming the Limitations of Sputtered Nickel Oxide for High‐Efficiency and Large‐Area Perovskite Solar Cells
Source: Adv Sci (Weinh). 2017 Oct 26;4(12):1700463. doi: 10.1002/advs.201700463 (PMC5737142; doi:10.1002/advs.201700463)
Supplement: Supplementary file 1 — Supplementary [file ADVS-4-na-s001.pdf]

Copyright WILEY-VCH Verlag GmbH & Co. KGaA, 69469 Weinheim, Germany, 2017.

ADVANCED SCIENCE
Open Access

Supporting Information

for *Adv. Sci.,* DOI: 10.1002/advs.201700463

Overcoming the Limitations of Sputtered Nickel Oxide for High-Efficiency and Large-Area Perovskite Solar Cells

Guijun Li, Yibin Jiang, Sunbin Deng, Alwin Tam, Ping Xu, Man Wong, and Hoi-Sing Kwok*

Copyright WILEY-VCH Verlag GmbH & Co. KGaA, 69469 Weinheim, Germany, 2016.

Supporting Information

Overcoming the Limitations of Sputtered Nickel Oxide for High-efficiency and Large-area Perovskite Solar Cells

Guijun Li, Yibin Jiang, Sunbin Deng, Alwin Tam, Ping Xu, Man Wong, and Hoi-Sing Kwok*

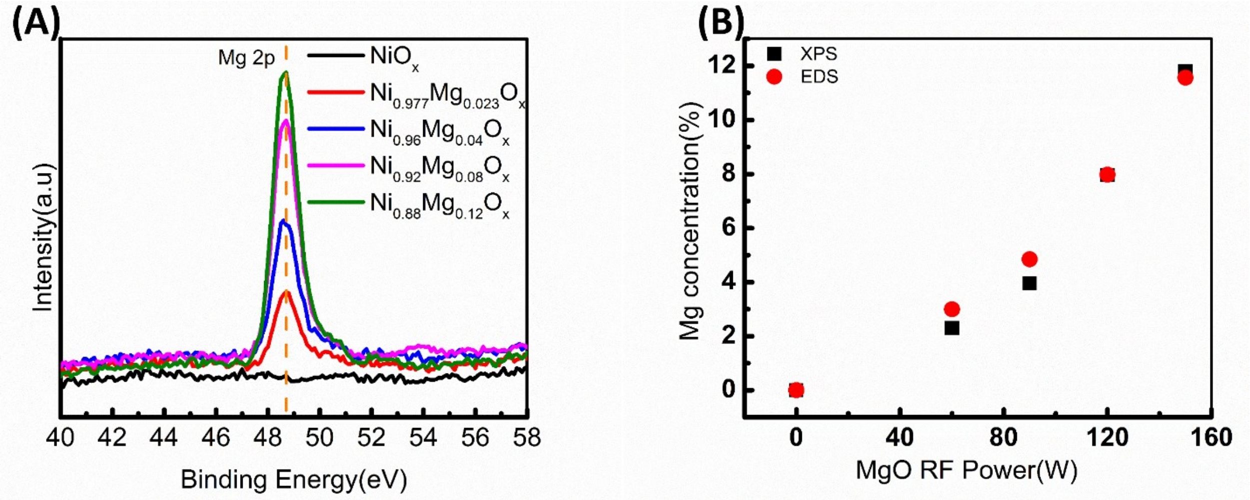

Figure. S1 (A) Mg 2p XPS spectra of films with different Mg concentrations. (B) Comparison of Mg concentration measured with XPS and EDS. Different concentrations are obtained by varying RF power of MgO during sputtering.

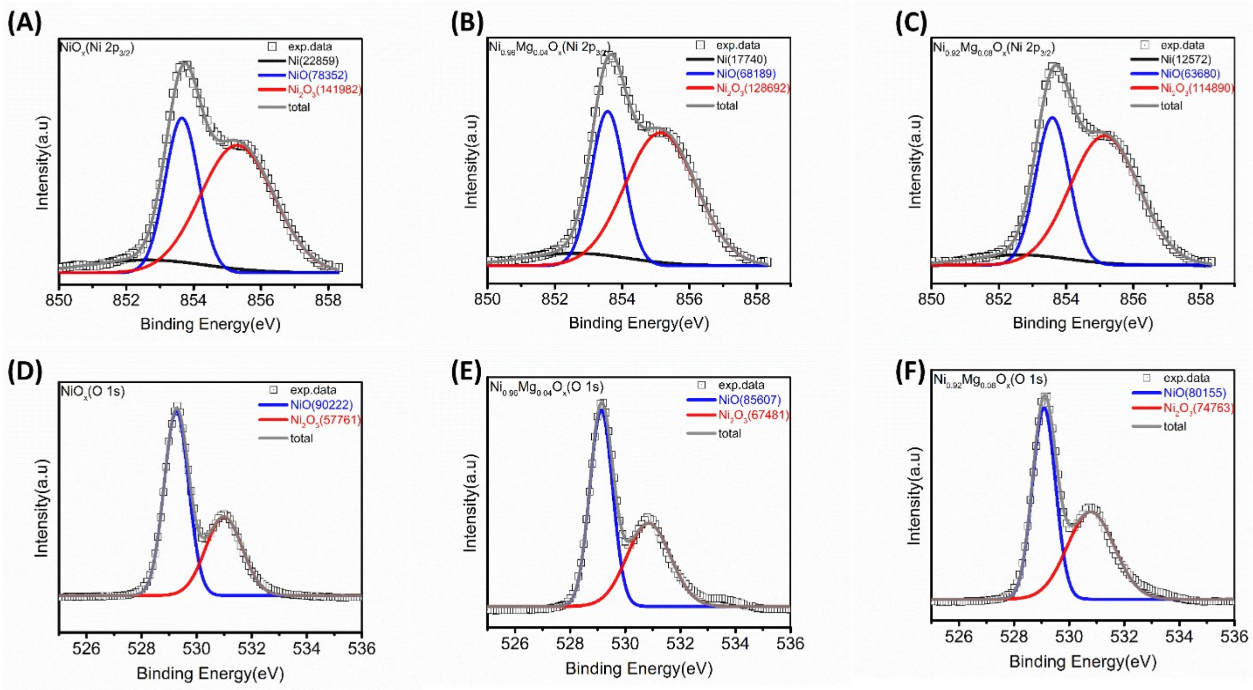

Figure. S2. Ni $2p_{3/2}$ spectrum for (A) NiO_x; (B) $Ni_{0.96}Mg_{0.04}O_x$; (C) $Ni_{0.92}Mg_{0.08}O_x$. O 1s spectrum for (D) NiO_x; (E) $Ni_{0.96}Mg_{0.04}O_x$; (F) $Ni_{0.92}Mg_{0.08}O_x$. The integrated areas of the fitted peaks are denoted in the corresponding brackets showing that the relative content of Ni metal (from Ni $2p_{3/2}$ spectrum) is reduced and Ni^{3+}(from O 1s spectrum) is increased upon Mg doping.

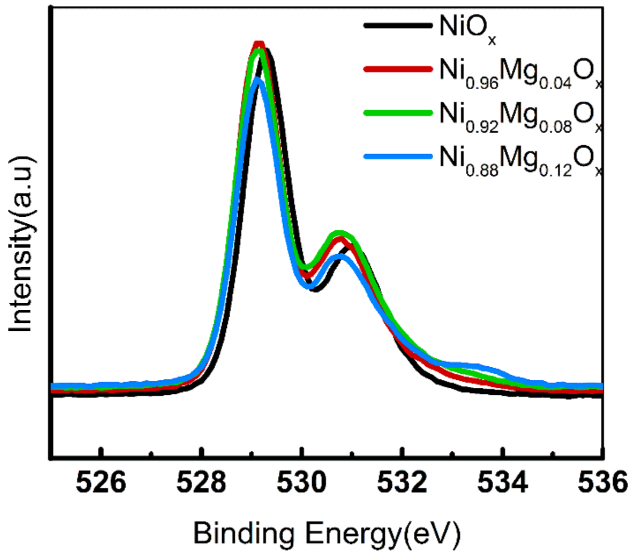

Figure. S3 O 1s spectra of different films. The peak positions shift to lower binding energy upon Mg doping.

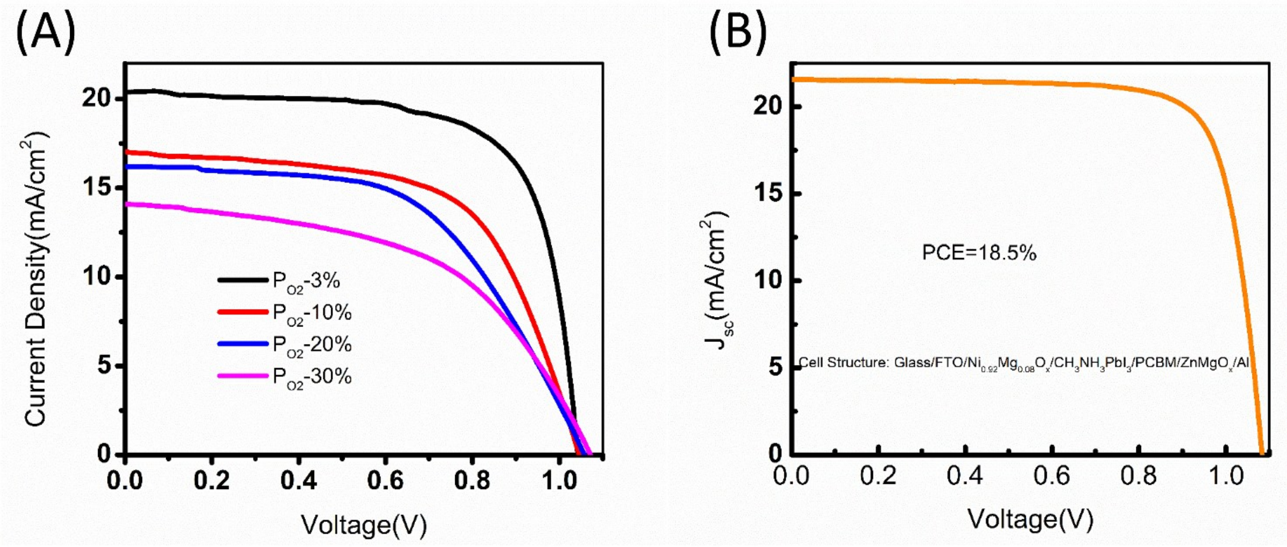

Figure. S4 (A) J-V curves of perovskite solar cells with NiO_x hole transport layers deposited at different oxygen partial pressures. The highest performance perovskite solar cells are obtained with the lowest oxygen partial pressure of 3%. (B) The J-V curve of the best perovskite solar cells with a $Ni_{0.92}Mg_{0.08}O_x$ hole transport layer. Data is collected with forward scan at a delay time of 150 ms.

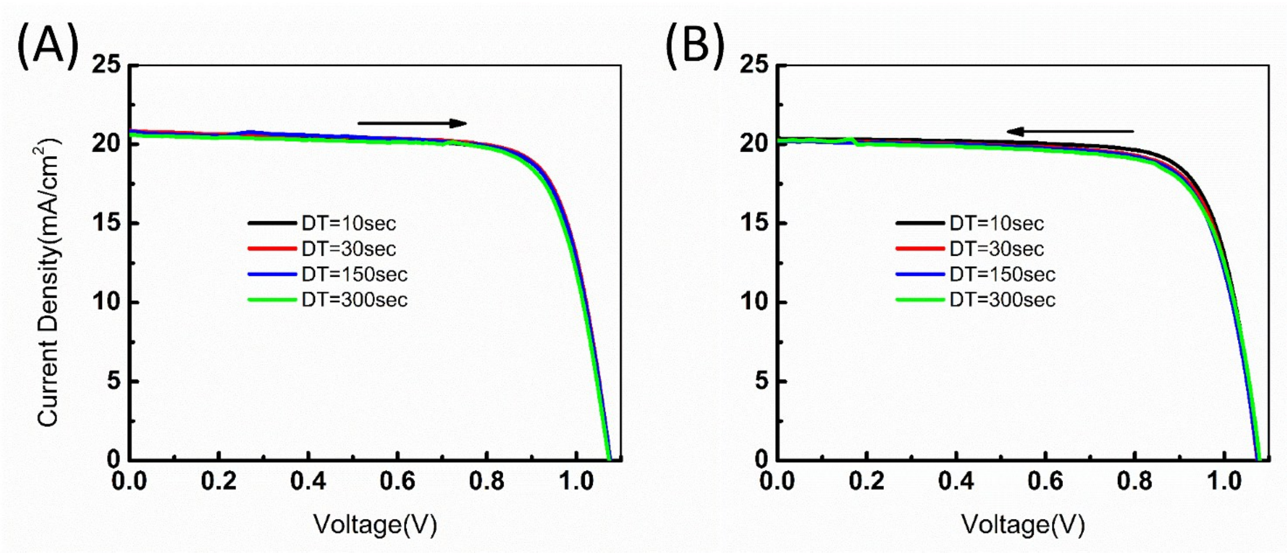

Figure. S5 The J-V curves of one of the high-performance $NiMgO_x$-based devices at different scan rates. (A) Forward sweep; (B) Reverse sweep. “DT” is the delay time required to measure the current at a given voltage. Reverse scan defines a measure from the open-circuit to the short-circuit and forward scan is vice versa.

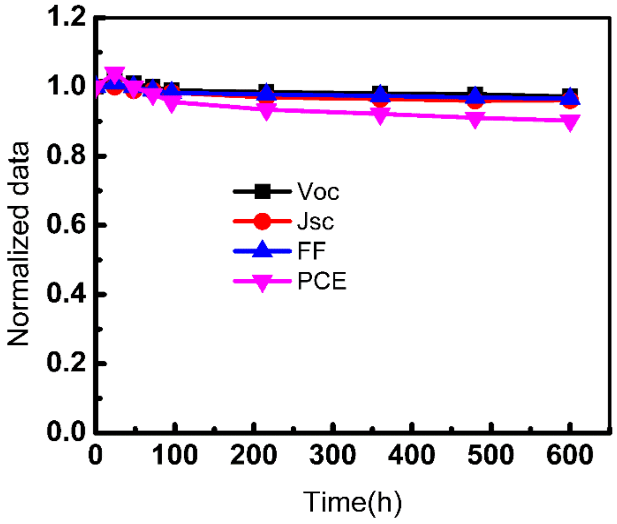

Figure. S6 The normalized data for PCE, V_{oc}, J_{sc}, and FF showing the ambient stability of the perovskite solar cells. The perovskite solar cells are encapsulated between two glasses with the epoxy. At the point of 24 hours, the PCE increases about 4% due to the increase of the V_{oc} and FF, which is believed to be the fact that ZnMgO has better contact with PCBM and AL after aging.

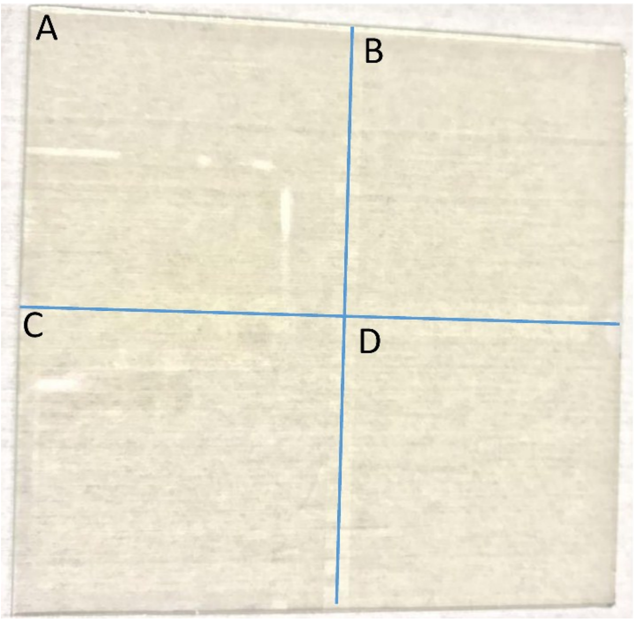

Figure. 7 the 10 cm x 10 cm substrate coated with NiMgOx. The NiMgOx has good uniformity over the size of 10 cm x 10 cm. Each substrate is divided into four sub-substrates A, B, C, and D.

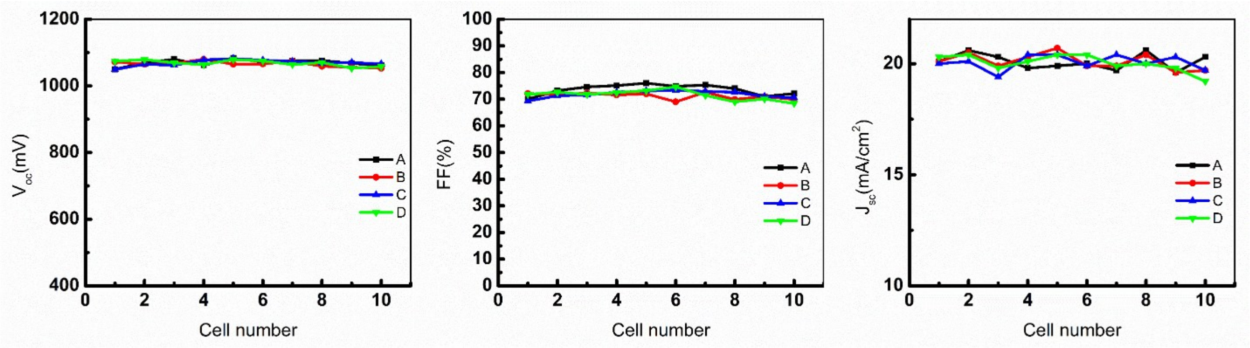

Figure. S8 the V_{oc}, FF and J_{sc} distributions. V_{oc} is almost constant for all the cells, where FF and J_{sc} have relatively large fluctuation.

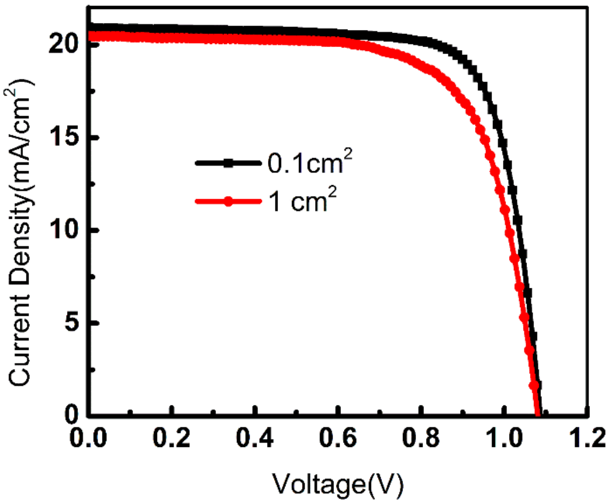

Figure. S9 J-V curves of perovskite solar cells with different cell areas.
